# Epigenetic regulation of bud dormancy events in perennial plants

**DOI:** 10.3389/fpls.2014.00247

**Published:** 2014-06-03

**Authors:** Gabino Ríos, Carmen Leida, Ana Conejero, María Luisa Badenes

**Affiliations:** ^1^Instituto Valenciano de Investigaciones AgrariasMoncada, Valencia, Spain; ^2^Research and Innovation Center, Fondazione Edmund Mach, San Michele all’AdigeItaly

**Keywords:** bud dormancy, chilling, *DAM* gene, chromatin, histone modifications

## Abstract

Release of bud dormancy in perennial plants resembles vernalization in *Arabidopsis thaliana* and cereals. In both cases, a certain period of chilling is required for accomplishing the reproductive phase, and several transcription factors with the MADS-box domain perform a central regulatory role in these processes. The expression of *DORMANCY-ASSOCIATED MADS-box* (*DAM*)-related genes has been found to be up-regulated in dormant buds of numerous plant species, such as poplar, raspberry, leafy spurge, blackcurrant, Japanese apricot, and peach. Moreover, functional evidence suggests the involvement of *DAM* genes in the regulation of seasonal dormancy in peach. Recent findings highlight the presence of genome-wide epigenetic modifications related to dormancy events, and more specifically the epigenetic regulation of *DAM*-related genes in a similar way to *FLOWERING LOCUS C*, a key integrator of vernalization effectors on flowering initiation in *Arabidopsis*. We revise the most relevant molecular and genomic contributions in the field of bud dormancy, and discuss the increasing evidence for chromatin modification involvement in the epigenetic regulation of seasonal dormancy cycles in perennial plants.

## SEASONALITY OF BUD DORMANCY FOR ADAPTATION

The vegetative and reproductive meristems of many perennial plants in temperate climates remain in a non-growing latent state during the cold period of autumn and winter, within buds, which ensure an optimal protection against low temperatures and drought. The cessation of meristem growth and bud set are controlled by photoperiod, temperature, or a combination of both in different species (Olsen, 2010; Cooke et al., 2012); followed by the induction of bud dormancy, which precludes the growth resumption even under favorable environmental conditions (Rohde and Bhalerao, 2007). Bud dormancy is dynamically modulated by intrinsic, environmental, and hormonal factors (Arora et al., 2003; Horvath et al., 2003; Allona et al., 2008; Cooke et al., 2012), which in several aspects, resemble those factors regulating dormancy in seeds (Powell, 1987; Leida et al., 2012b). Paradoxically, in species from the *Rosaceae* family among others, bud dormancy is released by the same low-temperature conditions that induce dormancy, differing in the cumulative and quantitative perception of chilling required for an effective dormancy release (Coville, 1920; Couvillon and Erez, 1985).

The genotype specificity of chilling requirements for dormancy release, in addition to fruit maturation dates, are two major factors limiting the geographical distribution of temperate species (Chuine, 2010). Under a perspective of global warming, phenological issues may have a critical impact on the distribution and performance of cultivars and species. For instance, a later fulfillment of the chilling requirements of the meadow and steppe vegetation of the Tibetan Plateau from the mid-1990s, due to a warmer environment, has been found associated with a delay in the beginning of the growing season (Yu et al., 2010). The length of the growth season, limited by the growth cessation and bud-burst dates, is nevertheless expected to increase or decrease in different species according to their particular interaction with the environment (Hänninen and Tanino, 2011). This significant impact on yield from established orchards could alter the range of both native and invasive plants, and it also represents a challenge for tree breeders involved in improving varieties for better adaptability to a particular environment.

## REGULATORY FACTORS IMPINGING ON BUD DORMANCY

The study of the mechanisms involved in bud dormancy initiation and release, and chilling perception, has been approached by genetic, genomic, and physiological analyses. Numerous transcriptomic studies have arisen during the last few years, addressing the changes in gene expression triggered by bud dormancy events in poplar (*Populus* spp.; Rohde et al., 2007; Ruttink et al., 2007), raspberry (*Rubus idaeus*; Mazzitelli et al., 2007), leafy spurge (*Euphorbia esula*; Horvath et al., 2008), Japanese apricot (*Prunus mume*; Yamane et al., 2008; Zhong et al., 2013), grapevine (*Vitis* spp.; Mathiason et al., 2009; Díaz-Riquelme et al., 2012), peach (*Prunus persica*; Jiménez et al., 2010a; Leida et al., 2010), blackcurrant (*Ribes nigrum*; Hedley et al., 2010), white spruce (*Picea glauca*; El Kayal et al., 2011), and pear (*Pyrus pyrifolia*; Liu et al., 2012; Bai et al., 2013) among other perennial species. These studies highlight the existence of common and specific gene expression programs affecting cell cycle regulation, light perception, hormonal signaling and stress response, and novel putative regulators of the dormancy stage of buds (Horvath et al., 2003; Cooke et al., 2012; Leida et al., 2012a; Doğramaci et al., 2013).

The identification and characterization of non-dormant mutants, such as the *evergrowing* (*evg*) mutant of peach (Rodriguez et al., 1994), in addition to several functional studies using transgenic poplar, have contributed to renew the dormancy field with increasing molecular works at the gene level. A deletion affecting several members of a series of six tandemly repeated MADS-box genes (*DAM1*-*6*, for *DORMANCY-ASSOCIATED MADS-box*) has been proposed to cause the non-dormant phenotype of the *evg* mutant (Bielenberg et al., 2004,^2008^). The six *DAM* genes of peach were presumably originated by serial tandem duplications from an ancestor related to the flowering transition regulator *SHORT VEGETATIVE PHASE* (*SVP*) of *Arabidopsis thaliana* (Jiménez et al., 2009). In addition to their tight linkage to the *evg* locus, several recent molecular studies support the involvement of *DAM* genes in promoting dormancy. *DAM* genes are expressed in buds following different developmental patterns and are distinctly affected by photoperiod and chilling signals (Li et al., 2009). However, the expression of two of them, *DAM5* and *DAM6*, correlates particularly well with the dormancy status of buds, being high in dormant buds and low after the fulfillment of chilling requirements prior to bud dormancy release (Jiménez et al., 2010b; Yamane et al., 2011). To acquire further genetic evidences about *DAM* function, *DAM6* has been constitutively expressed in transgenic plums (*Prunus domestica*), showing some degree of dwarfing and increased branching (Fan, 2010). Moreover, several independent quantitative trait loci (QTL) analyses dissecting the flowering time and chilling requirement traits in peach (*P. persica*), apricot (*Prunus armeniaca*), and almond (*Prunus dulcis*) have found a common QTL in linkage group 1 coincident with the genomic location of the *DAM* locus (Quilot et al., 2004; Olukolu et al., 2009; Fan et al., 2010; Sánchez-Pérez et al., 2012; Romeu et al., 2014). Interestingly, the low chilling requirement trait evaluated in one of these QTL segregating populations in peach, is associated with the presence of large insertions within intronic sequences in *DAM5* and *DAM6* genes (Zhebentyayeva et al., 2014). However, none of the poplar homologs of *DAM* genes colocalizes with QTLs associated with bud set in this species (Rohde et al., 2011), suggesting a somehow different role or a reduced natural variability of *DAM* genes in poplar.

Related *DAM*-like genes with dormancy-dependent expression have been found in other perennial species such as raspberry (Mazzitelli et al., 2007), leafy spurge (Horvath et al., 2008,^2010^), Japanese apricot (Yamane et al., 2008), pear (Ubi et al., 2010; Saito et al., 2013), blackcurrant (Hedley et al., 2010), and kiwifruit (*Actinidia deliciosa*; Wu et al., 2012). The heterologous expression of some of these genes in transgenic plants has offered additional clues about their role in flowering and dormancy. *Arabidopsis* plants expressing *DAM1* from leafy spurge (Horvath et al., 2010) and *SVP1* and *SVP3* from kiwifruit (Wu et al., 2012) show a delay in flowering time; whereas the expression of *PmDAM6* from Japanese apricot in poplar causes growth cessation and bud set under conditions favorable for growth (Sasaki et al., 2011).

Other regulatory genes belonging to different clades within the MADS-box family have also been found to be related to bud dormancy events. Thus, the overexpression of the birch (*Betula pendula*) *FRUITFULL* (*FUL*)-like gene *BpMADS4* delays senescence and winter dormancy in *Populus tremula* (Hoenicka et al., 2008), and the presence of different allelic variants in a *SUPPRESSOR OF OVEREXPRESSION OF CONSTANS (CO) 1* (*SOC1*)-like gene in apricot is found associated with different chilling requirements for dormancy break (Trainin et al., 2013). Interestingly, *FUL* and *SOC1* are activated in the shoot apex of *Arabidopsis* by the mobile florigen protein produced by the *FLOWERING LOCUS T* (*FT*) gene, and mediate the effect of *FT* and *CO* on the photoperiod-dependent induction of flowering (Yoo et al., 2005; Melzer et al., 2008). In poplar, a regulatory pathway including two genes homologous to *CO* and *FT* controls the growth cessation induced by short days, and also seasonal bud set (Böhlenius et al., 2006). Moreover, transgenic plums overexpressing *FT1* from poplar do not enter dormancy and experience continuous flowering among other developmental alterations (Srinivasan et al., 2012). A deep study about the role of the paralogs *FT1* and *FT2* in poplar, using heat-inducible constructs, has shown that *FT1* determines the reproductive onset in winter, whereas *FT2* promotes the vegetative growth in spring and summer (Hsu et al., 2011). This work postulates the seasonal succession of reproductive, vegetative, and dormant phases in poplar through the divergent expression and function of *FT1* and *FT2* genes.

The role of abscisic acid (ABA) and ABA-responsive factors in dormancy maintenance in buds has been mainly addressed in poplar. The overexpression and downregulation of *PtABI3*, an homolog of *ABSCISIC ACID INSENSITIVE 3* (*ABI3*) of *Arabidopsis* involved in seed dormancy regulation by ABA signaling, cause developmental alterations in bud formation and misregulation of gene expression during bud dormancy processes (Rohde et al., 2002; Ruttink et al., 2007). Furthermore, the ectopic expression of the mutant gene *abscisic acid insensitive 1* (*abi1*) in poplar modifies the dormant response of buds to exogenous ABA (Arend et al., 2009).

## PIECES OF AN EPIGENETIC “ALARM CLOCK” FOR BUD DORMANCY AND AWAKENING

Several reviews postulate the participation of different epigenetic mechanisms involving histone modification, DNA methylation, and the synthesis of small non-coding RNAs in regulating bud dormancy events, based on evident similarities between the environmental and molecular control of dormancy in buds and other well-known processes, such as flowering initiation, vernalization, and seed development (Horvath et al., 2003; Horvath, 2009; Hemming and Trevaskis, 2011; Cooke et al., 2012). However, only few recent works have provided experimental support for these postulates.

**FIGURE 1:**
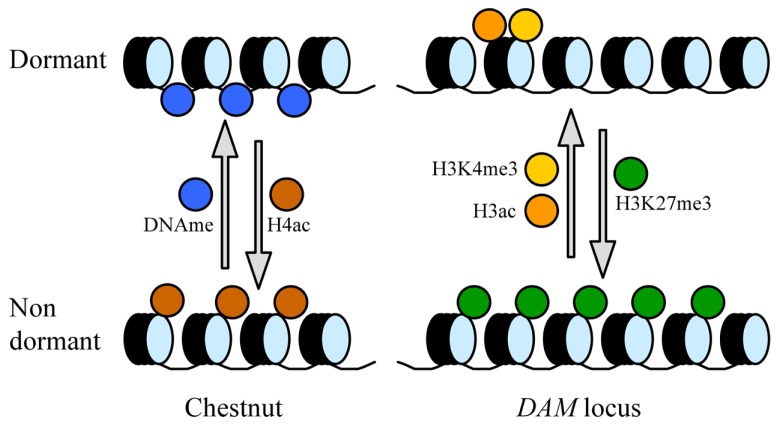
**General and specific modifications of chromatin in dormant and dormancy-released buds**. The following chromatin modifications have been identified in chestnut at the genome level (left), and specifically in the *DAM* locus in leafy spurge and peach (right): DNA methylation (DNAme), acetylation of histone H4 (H4ac), acetylation of H3 (H3ac), trimethylation of H3 at K4 (H3K4me3), and trimethylation of H3 at K27 (H3K27me3).

The global levels of genomic DNA methylation and acetylated histone 4 (H4) show cyclic and opposite variations during the seasonal development of chestnut (*Castanea sativa*), with higher DNA methylation ratios and lower H4 acetylation levels in dormant buds with respect to actively growing tissues (Santamaría et al., 2009; **Figure 1**). These data support a significant silencing of bulk gene expression concomitant with bud dormancy; however, the epigenetic regulation of particular genes with a relevant role in regulatory issues may differ from this global tendency to gene repression. Indeed, in a subsequent work, the authors have found the gene *CsAUR3* encoding an H3 kinase-like expressed in growing tissue, and genes *CsHUB2* and *CsGCN5L* coding for putative histone mono-ubiquitinase and histone acetyltransferase with higher expression in dormant buds (Santamar໚ et al., 2011). Interestingly, the *hub2* mutant in *Arabidopsis* displays reduced seed dormancy (Liu et al., 2007). In agreement with overall DNA methylation changes in chestnut, a decrease in DNA methylation at 5′-CCGG-3′ sites precedes dormancy release, transcriptional activation, and meristem growth in potato tubers (Law and Suttle, 2003).

Some transcriptomic studies have contributed to the identification of genes involved in epigenetic regulation differentially expressed in dormant and growing samples. Two genes similar to *FERTILIZATION INDEPENDENT ENDOSPERM* (*FIE*) and *PICKLE* (*PKL*) are strongly up-regulated in poplar immediately after transfer to short-day (SD) conditions, leading to bud formation and dormancy induction (Ruttink et al., 2007). FIE is a component of the Polycomb repressive complex 2 (PRC2) involved in the trimethylation of H3 lysine 27 (H3K27me3), a chromatin modification associated with stable gene silencing, whereas PKL is an ATP-dependent chromatin-remodeller that regulates embryo identity traits and associates with multiple H3K27me3-enriched loci (Ogas et al., 1999; Zhang et al., 2012). In *Arabidopsis*, *fie* mutants show fertilization-independent development of the endosperm (Ohad et al., 1999), precocious flower-like structures (Kinoshita et al., 2001), and seeds with impaired dormancy (Bouyer et al., 2011). According to these data suggesting a role of Polycomb complexes in SD-induction of dormancy, transgenic hybrid aspen lines with RNAi-mediated downregulation of *FIE* are not able to establish dormancy, even though growth cessation and bud formation processes seem not to be affected (Petterle, 2011).

Other genes up-regulated during the activity–dormancy transition in hybrid aspen encode putative histone deacetylases (*HDA14* and *HDA08*), histone lysine methyltranferase (*SUVR3*), and *HUB2*, while several genes belonging to the Trithorax family of factors counteracting the repressive effect of Polycomb complex and putative *DEMETER*-like DNA glycosylases are down-regulated during the same period (Karlberg et al., 2010). These results prompted the authors to propose a model in which some unknown target genes are repressed by chromatin compaction during dormancy induction due to histone deacetylation and methylation, histone ubiquitination, DNA methylation, and Polycomb activity. Interestingly, dormancy release upon chilling treatment is accompanied by the up-regulation of other histone deacetylase genes (*HDA9* and *SIN3*) and also a *DEMETER*-like gene, suggesting that chromatin repression mechanisms with distinct target specificity could act on the different stages of the activity–dormancy cycle (Karlberg et al., 2010).

## RESEMBLANCES OF BUD DORMANCY AND VERNALIZATION

The similarities of bud dormancy in perennials with the vernalization treatment for flowering in *Arabidopsis* and cereals reside in their common requirement of a time period of quantitative and cumulative chilling, and the participation of certain MADS-box domain transcription factors with key regulatory tasks (Chouard, 1960; Horvath et al., 2003; Hemming and Trevaskis, 2011). The MADS-box genes *FLOWERING LOCUS C* (*FLC*) and *VERNALIZATION1* (*VRN1*) control, respectively, the vernalization response in *Arabidopsis* and cereals (Sheldon et al., 2000; Trevaskis et al., 2007), whereas *DAM* genes exert an analogous role in bud dormancy processes. Interestingly, the vernalization and bud dormancy responses are mediated by the regulation of *FLC*, *VRN1*, and *DAM* gene expression through common epigenetic mechanisms. After prolonged cold exposure, concomitantly with gene repression and dormancy release, the chromatin in the promoter of leafy spurge *DAM1* shows a decrease in trimethylation of H3 lysine 4 (H3K4me3) and an increase of H3K27me3 (Horvath et al., 2010), two histone modifications found also associated with the vernalization-dependent repression of *FLC* in *Arabidopsis* (Bastow et al., 2004; Kim et al., 2005), and in *VRN1* in barley before vernalization (Oliver et al., 2009). In peach, *DAM6* shows similar epigenetic changes associated with gene repression after dormancy release, in addition to a decrease of H3 acetylation in the chromatin around the ATG region (Leida et al., 2012a; **Figure 1**). Such changes occurred at different dates in two different cultivars, in close agreement with their particular chilling requirements and dormancy release dates (Leida et al., 2012a).

In addition to histone modifications, the synthesis and action of long non-coding RNAs (lncRNAs) play a crucial role in the epigenetic regulation of *FLC* gene in *Arabidopsis* (Turck and Coupland, 2011). Both, a natural antisense transcript called *COOLAIR* expressed from a promoter located in the 3′ flanking region of *FLC*, and a sense intronic lncRNA named *COLDAIR*, participate in different phases of the cold-induced repression and the stable silencing of *FLC* by PRC2 (Swiezewski et al., 2009; Heo and Sung, 2011).

Other non-coding RNAs acting on the epigenetic regulation of many plant processes are microRNAs (miRNAs). Recently, a genome-wide approach for the isolation of conserved and novel peach miRNAs has allowed the identification of miRNAs differently expressed in chilled vegetative buds (Barakat et al., 2012). Some of these miRNAs colocalize with known QTLs for chilling requirement and blooming date traits, offering new tools and targets for the genetic analysis of dormancy mechanisms. An epigenetic mechanism involving the synthesis of specific miRNA has been also proposed for an adaptive memory of temperature observed in Norway spruce, in which the environmental temperature during embryo development determines the bud phenology and cold acclimation (Yakovlev et al., 2010,^2011^). An miRNA cascade involving miR156 and miR172 and their respective targets *SQUAMOSA PROMOTER BINDING PROTEIN-LIKE* (*SPL*), and *APETALA2* (*AP2*)-like genes modulates flowering induction in *Arabidopsis* through the regulation of *FT* and other flowering-related genes (Khan et al., 2014; Spanudakis and Jackson, 2014). Evidence on the differential expression of *SPL* genes and miR172 during dormancy induction has been obtained from transcriptomic studies in poplar and leafy spurge (Ruttink et al., 2007; Doğramaci et al., 2013), which suggests that this miRNA pathway may also play a regulating role in dormancy processes.

## PERSPECTIVES

As new details of the epigenetic regulation of gene expression during bud dormancy processes emerge, its mechanistic similarities with the well-known regulation of *Arabidopsis FLC* gene are becoming increasingly evident. Based on such analogy, one may reasonably figure out the remaining pieces of the “alarm clock” outlined above. The chromatin of the transcription start site of pre-vernalized *FLC* is trimethylated at H3K36 and monoubiquitinated at H2B, favored by the interaction of chromatin modification enzymes with the transcriptional machinery (He, 2012). These modifications and proteins could be similarly identified in dormant buds of perennial species where *DAM*-like genes are transcriptionally active, prior to cold-dependent down-regulation. Also cold-induced *COOLAIR*-like and *COLDAIR*-like lncRNAs could be involved, respectively, in the initial down-regulation of *DAM* genes and the function of PRC2 complexes. In fact, the presence of a large intron shortly after the beginning of the coding region in *DAM* genes in peach resembles the situation of the first intron of *FLC*, particularly important in the synthesis of *COLDAIR* and the nucleation of the PRC2-dependent trimethylation of H3K27 (Song et al., 2012). In spite of the coincident identification of H3K27me3 in the chromatin of *DAM*-like genes in dormancy-released buds of leafy spurge and peach, the components and the role of PRC2 complexes in the stable silencing of *DAM* need to be established in temperate perennials, as already performed in *Arabidopsis* (Jiang et al., 2008). In summary, the rich literature describing vernalization in model plants may radically accelerate the knowledge of the epigenetics of bud dormancy regulation over the next few years, but it will require the implementation of more informative biochemical and functional approaches in perennial species to complement previous genetic and genomic studies.

In addition to these mechanistic commonalities between the regulation of vernalization and bud dormancy, specific regulatory elements are expected to operate in buds. In particular, the seasonality of bud dormancy with undefined repeated cycles of growth–dormancy along the life of an individual, the major role of hormones such as ABA, and the occurrence of dormancy-related processes on preformed flowers located within buds instead of vegetative tissues, among other particularities, suggest the existence of yet unknown exciting pieces in the “clock.”

## Conflict of Interest Statement

The authors declare that the research was conducted in the absence of any commercial or financial relationships that could be construed as a potential conflict of interest.
